# Commercial conspiracy theories: a pilot study

**DOI:** 10.3389/fpsyg.2013.00379

**Published:** 2013-06-27

**Authors:** Adrian Furnham

**Affiliations:** Research Department of Clinical, Educational, and Health Psychology, University College LondonLondon, UK

**Keywords:** commercial conspiracy theories, politics, Big Five, individual differences

## Abstract

There are many ways to categorise conspiracy theories. In the present study, we examined individual and demographic predictors of beliefs in *commercial conspiracy* theories among a British sample of over 300 women and men. Results showed many people were cynical and sceptical with regard to advertising tricks, as well as the tactics of organisations like banks and alcohol, drug and tobacco companies. Beliefs sorted into four identifiable clusters, labelled sneakiness, manipulative, change-the-rules and suppression/prevention. The high alpha for the overall scale suggested general beliefs in commercial conspiracy. Regressions suggested that those people who were less religious, more left-wing, more pessimistic, less (self-defined as) wealthy, less Neurotic and less Open-to-Experience believed there was more commercial conspiracy. Overall the individual difference variables explained relatively little of the variance in these beliefs. The implications of these findings for the literature on conspiracy theories are discussed. Limitations of the study are also discussed.

## Commercial conspiracy theories

Conspiracy theories are beliefs that attribute the ultimate cause or concealment of an event or behavioural pattern from public knowledge, to secret, unlawful, and malevolent plots or processes, usually by multiple actors working together (Zonis and Joseph, 1994). Beliefs in conspiracy theories are widespread across the globe (Hofstadter, 1965; Moynihan, 1985; Graumann and Moscovici, 1987; Goertzel, 1994; Robins and Post, 1997; Byford and Billig, 2001; Byford, 2011; Swami et al., 2011), although they appear to be prominent in the West, particularly in America where the film industry has been implicated in spreading theories in the Cold War and the McCarthyism periods. It is also there that most commercial conspiracy theories appear to originate and thrive, especially those concerned with subliminal advertising (Crook, 2004). As a consequence there are numerous books that warn people about how advertisers and retailers attempt to “subliminally” influence people, such as that by Howard (2005) with the subtitle “The secret tactics that influence what you buy, think and believe.”

There are many ways to codify or classify conspiracy theories: event based vs. systemic; past vs. present vs. future; inside vs. outside forces; natural vs. supernatural; idiosyncratic vs. shared, as well as area specific: i.e., religious, political, health or commercial. This study will examine conspiracy theories concerned with business and commercial organizations that are seen to use devious, hidden and possibly illegal methods to persuade people to buy their products. It appears as if there is very little research in this area and this pilot study hopes to encourage more work in this neglected topic of conspiracy research.

Those who share conspiracy theories argue that it is usually naïve to believe in the official version of events because governments and corporations are Machiavellian manipulators of the media who often try to keep people in a state of ignorance and fear. Those with a “cover-up” as opposed to a “conspiracy theory” mindset accuse others of demonising them and being close-minded, whereas what they are doing is actually holding those in power to account and reclaiming history. They are often motivated by strong socio-political and religious ideologies (Byford, 2011).

It has been argued that conspiracy theories are psychologically functional in that they help individuals attain or maintain a sense of meaning, control and personal security (Newheiser et al., 2011). Miller (2002) suggests that conspiracy theories fulfil two essentially cognitive roles: an argumentative role and a social critique role. Shermer (2010) argued that in general conspiracy theories are held by people with four traits: *patternicity* (the tendency to find meaningful patterns in random noise), *agenticity* (the beliefs that the world is controlled by an/many invisible, intentional agent(s); *confirmation bias* (the strong preference to seek/find conformational evidence for what they believe) and *hindsight bias* (tailoring after-the-fact explanations to what they already know happened).

This study is on commercial conspiracy theories. There are many theories in this area, though most involve marketing tricks (Jacobson and Mazur, 1995) or more generally subliminal influences in shops, the media and the web (Howard, 2005). A great deal of this work concerns advertising (Crook, 2004) and the debate as to the efficacy of subliminal advertising on radio and television (Verwijmeren et al., 2011; Legal et al., 2012). There are many web-based sites which suggest that drug, food, and energy companies take part in massive and frequent cover-ups concerning their advertising and the information that they spread. Others are very product and event specific, such as the introduction and subsequent withdrawal of New Coke by the Coca Cola company (Hays, 2004). There are books designed to alert or inoculate people against commercial tactics such as that by Howard (2005).

### Psychological literature

Until recently there were few books with a psychological perspective on conspiracy theories (Byford, 2011). Some early works trace beliefs in conspiracy theories to feelings of powerlessness, particularly among marginalised people who believe they have become voiceless (Hofstadter, 1965). Various studies have shown that conspiracy theories are indeed associated with political cynicism, authoritarianism and support for democratic principles (Swami et al., 2010; Swami, 2012; Swami and Furnham, 2012).

Other early work suggested that beliefs in conspiracy theories serve self-esteem maintenance purposes (Young, 1990; Robins and Post, 1997), while providing believers an outlet for reasserting their individualism (Melley, 2000) or for the expression of negative feelings (Hofstadter, 1965; Ungerleider and Wellisch, 1979). Recently Swami and Furnham (2012) found conspiracy beliefs in one famous story- the disappearance of Amelia Earheart- were associated with lower self-esteem and lower self-estimated intelligence.

Few studies have attempted to examine the individual difference correlates of beliefs in conspiracies (Goertzel, 1994; Abalakina-Paap et al., 1999; Crocker et al., 1999). In general, these studies showed that belief in conspiracist ideas were correlated with anomia, low levels of interpersonal trust, feelings of social and political alienation, and perceptions of being disadvantaged. Some more recent studies have shown conspiracist beliefs related to the personality disorders (schizotypy) (Darwin et al., 2011) and also to personality. Studies using the Big Five personality traits have tended to show small but significant associations between two traits- Agreeableness and Openness- and beliefs in conspiracy theories (Swami et al., 2010, 2011).

Overall the psychological studies on individual difference correlates of specific conspiracy theories have confirmed various hypotheses but have also shown that any or all of the variables identified have been very modestly related to the endorsement of the theories accounting in total for less than around a fifth of the variance (Swami et al., 2010, 2011).

### The present study

The present study was conceived as a preliminary attempt to investigate correlates of some commercial conspiracy theories. In the present study, the focus was on personality and ideological variables, in the expectation that general psychological traits allow for the construction of a profiling model of conspiracist individuals. Specifically the study examined (among a British sample) the association of 30 commercial conspiracy beliefs, the “Big Five” personality factors, and personal ideology as defined by beliefs in politics, religion, self-perceived wealth and optimism/pessimism. Two hypotheses guided the selection of variables in the present work. First, it was expected that politically left-wing, less religious, and more pessimistic people would be more likely to endorse commercial conspiracy theories. In many studies of people who chose alternative and complementary medicine and were suspicious of, and cynical about, orthodox medicine, Vincent and Furnham (1997) found two simple questions concerning political and religious beliefs to consistently account for reasonable amounts of variance. The less religious and more politically left wing seemed most distrustful of orthodox medicine and its dependence on drug companies, and were instead in favour of alternative therapies. Second, it was expected that low Agreeableness and high Openness scores would be significantly associated with commercial conspiracy theories as various studies have shown these associations (Swami et al., 2010, 2011).

## Method

### Participants

Three hundred and twenty four individuals took part in the present study, of which 214 were women. Their average age was 23.42 years (*SD* = 10.16) ranging from 18 to 65. Just over half were young people in education and the remainder came from a variety of occupations. Asked how religious they were (1 = “Not at all” to 10 = “Very”) they scored 4.14 (*SD* = 2.82); their political orientation (1 = “Strongly Right Wing” to 10 = “Strongly Left Wing”) scored 5.51 (*SD* = 1.66); their personal optimism (1 = “Optimistic” to 10 = “Pessimistic”) was 4.26 (*SD* = 2.12) and Self-perceived wealth (1 = “Rich” to 10 = “Poor”) 5.02 (*SD* = 1.64).

### Measures

#### Belief in commercial conspiracy theories inventory

This is a 30-item, novel questionnaire devised for this study. The limited literature was consulted and over 60 potential items written. These were obtained from various websites but also books on anti-commercialism and advertising tactics (Jacobson and Mazur, 1995; Howard, 2005). These initial items were *Q* sorted by two people to get some idea of the categories the items were covering. They were then given to 10 people to complete with the instructions to be highly critical of the clarity of the statements and response scale. Those items that were unclear as well as those that showed floor and ceiling effects were discarded. Floor and ceiling effects were defined by the mean score suggesting that nearly all people in the pilot study either thought these activities virtually never (mean score >5.5) or very regularly (mean score <1.5) occurred. This left 30 items, which can be seen in Table 1. Participants are required to rate how regularly (1 = Very to 6 = Never) a number of practices they believed occurred.

**Table 1:** **Means and Standard Deviations for each of the 30 items**.

**No.**	**Statement**	**Mean**	***SD***
1	Placing the word “sex” very subtly in advertisements to attract your attention	3.10	1.35
2	Advertisers flashing subliminal (below consciousness) images in television advertisements	3.44	1.37
3	Supermarkets using undetectable gasses (smells) in shops to change a person's mood to encourage sales	3.96	1.52
4	Advertisers targeting specific audiences through their choice of timing of advertisements in programs	1.69	1.01
5	Shops tricking you with pricing: i.e., putting up prices for a few minutes, then down claiming big discounts	2.43	1.42
6	Junk mailers using “sneaky tactics” to get people to open the envelope	2.52	1.42
7	Advertisers disguising their ads in envelopes appearing to be official government documents	3.75	1.53
8	Alcohol and tobacco companies sponsoring various events to appeal to particular groups	2.61	1.49
9	Drug companies falsifying their data on the effectiveness of their drugs	3.45	1.41
10	Drug companies bribing doctors with presents and conferences to prescribe their drugs	3.50	1.60
11	Tobacco companies actually approving of cigarette smuggling	4.09	1.40
12	Tobacco companies trying to get around the advertising laws in every country	3.00	1.43
13	Various companies (mining, tobacco, drug) bribing politicians in any country they can to get laws passed to protect them	3.12	1.40
14	Drug companies torturing millions of animals in trials	3.08	1.43
15	Companies selling medically prescribed drugs which they know are addictive	2.91	1.37
16	Oil Companies deliberately suppressing better car technology that uses less fuel	3.29	1.31
17	Oil companies encouraging politicians to invade countries to take their oil	3.69	1.45
18	Manufacturers using copy-cat product packing to trick shoppers into buying more	2.50	1.23
19	Light bulb companies preventing technological advances into producing longer-lasting light bulbs	3.64	1.36
20	Governments banning certain third world product not because they are unsafe but because they complete too well	3.26	1.37
21	Government guidelines setting poor diet guidelines so that the medical industry generates drug and treatment revenue in unhealthy patients	4.32	1.37
22	Oil companies intentionally ignoring oil reserves to create the illusion of scarcity that keeps prices high	3.33	1.37
23	Manufacturers adding Illegal additives to foods (i.e., some brand of crisps) to make them addictive	3.45	1.51
24	Effective alternative medicines being rejected by medical councils to maximize revenue	3.50	1.40
25	Drug companies getting normal behaviour being called a *disorder* so they can invent drugs to cure it	3.74	1.45
26	Food companies being dishonest about genetically modified food	3.03	1.34
27	Banks manipulating inflation and other figures to make more profit	2.93	1.46
28	Lawyers knowing lying on behalf of their clients	2.21	1.18
29	Shops faking “sell-by” dates to make more profit	3.24	1.49
30	Jews working in high-power jobs in the media spreading propaganda to gain support for Israel	4.13	1.49

*Scale: 6 = Never to 1 = Very regularly*.

*Abbreviated, 15-item Big Five Questionnaire* (e.g., Furnham et al., 2003). This is a brief scale for assessing the Big Five personality factors, suitable for looking at population-level correlations. It was chosen in this study because it yields acceptably reliable scores yet is very brief. The five personality factors were arrived at by summing certain items, and alpha coefficients were as follows: Openness α = 0.57, Conscientiousness α = 0.52, Extraversion α = 0.60, Agreeableness α = 0.5, and Neuroticism α = 0.67. These alpha coefficients are not ideal, but it should be remembered that they were calculated using three items each and that they are in line with population norms reported in previous work (e.g., Furnham et al., 2003).

### Procedure

Ethical committee permission was sought and received. Participants were approached in two settings: lectures to a variety of groups given by the author (where around 40% of the sample was obtained) and in two large London railway stations where people waiting were approached. The questionnaire took around 5 min to complete. Overall the response rate was around 80%. Where possible participants were thanked and debriefed after they had anonymously and voluntarily completed the questionnaire.

## Results

The results for the individual items are shown in Table 1. Three were seen to occur relatively regularly (items 4, 5, and 28), some relatively frequently (item 6, 8, 15, 18, and 27) and three rarely (items 11, 21, and 30).

Two attempts were made to investigate the underlying structure of the items. First, both oblique (promax) and orthogonal (varimax) rotated factor analytic statistics were calculated. Second a *Q*-sort test was done by two people trying to determine the content structure of the questionnaire. None of the factor analyses yielded clearly interpretable factors, though the *Q*-sort did. Both had five categories. Only where there was internal agreement that items belong in the same category were they retained and this left four categories. Four sets of items were grouped together under four labels: *Sneakiness* (items 1, 4, 5, 6, 7, 8, 18, 28); *Manipulative* (items 9, 10, 11, 14, 15, 23, 26, 29); *Changing the Rules* (items 12, 13, 17, 20, 21, 24, 27) and *Suppression/Prevention* (items 16, 19, 22, 25, 30). These four scales were intercorrelated—the highest being between factors 2 and 3 (*r* = 0.68) and the lowest being between factors 1 and 3 (*r* = 0.34). The alpha coefficients were then calculated for each factor in turn and they were: 0.64, 0.73, 0.69, and 0.63, respectively. The Alpha for the whole scale of 30 items was also calculated and this was 0.86, suggesting evidence of a monological commercial conspiracy belief system.

Next a correlation matrix was calculated between the gender, four personal ratings (religion, politics, optimism, and wealth), the big five personality factors and the four conspiracy factors and total conspiracy score. Few were significant. These are shown in Table 2.

**Table 2:** **Correlations between beliefs, personality and the factors**.

	**R**	**P**	**Opt**	**W**	**N**	**E**	**Ope**	**A**	**C**	**F1**	**F2**	**F3**
Religiosity (R)												
Politics (P)	−0.05											
Optimism (Opt)	0.18	−0.15										
Wealth (W)	0.01	0.00	0.28									
Neuroticism (N)	0.00	0.06	0.10	0.04								
Extraversion (E)	0.07	0.00	−0.17	0.03	−0.01							
Openness (Ope)	0.05	0.19	0.10	−0.02	−0.04	0.09						
Agreeableness (A)	−0.03	0.00	0.06	0.00	−0.10	0.11	0.06					
Conscientiousness (C)	0.06	−0.16	−0.11	−0.04	−0.08	0.08	0.00	−0.10				
Factor 1	0.09	0.07	0.09	0.02	0.08	−0.09	−0.05	−0.13	0.03			
Factor 2	−0.05	0.08	−0.03	−0.25	−0.04	0.00	−0.02	0.06	0.06	0.34		
Factor 3	−0.12	0.05	−0.07	−0.10	−0.10	−0.11	−0.07	0.13	0.13	0.34	0.68	
Factor 4	−0.08	0.01	0.00	−0.17	−0.11	0.02	−0.18	0.13	0.13	0.36	0.61	0.61

Finally a series of five regressions was performed with the four conspiracy factor scores and the total conspiracy score as the criterion variables and three sets of predictor variables: sex and age, the four self ratings and the Big Five factor scores. Three were significant. The regression for the second factor (Manipulative) was significant [*F*_(9, 235)_ = 2.23, *p* < 0.05, AdjR^2^ = 0.08] showing that those who rated themselves poorer (β = −0.28, *t* = 3.20, *p* < 0.001) believed more this more strongly. The fourth factor (Suppression/Prevention) was also significant [*F*_(9, 236)_ = 3.42, *p* < 0.001, AdjR^2^ = 0.14]. The results showed that those who endorsed this factor tended to be less religious (β = −0.19, *t* = 2.33, *p* < 05), more pessimistic (β = 0.23, *t* = 2.40, *p* < 0.01), poorer (β = −0.25, *t* = 2.99, *p* < 0.01), less Neurotic (β = −0.22, *t* = 2.67, *p* < 0.01) and less Optimistic (β = −0.24, *t* = 2.81, *p* < 0.01).

The total score regression was also significant [*F*_(9.229)_ = 2.19, *p* < 0.05, AdjR^2^ = 0.08). There were three significant predictors. Those who endorsed commercial conspiracies were politically left wing (β = 0.18, *t* = 1.94, *p* < 0.05), more pessimistic (β = 0.22, *t* = 2.12, *p* < 05) and rated themselves as poorer (β = −0.25, *t* = 2.73, *p* < 0.001). Two other variables narrowly missed significance: those who were less religious (β = −0.15, *t* = −1.70, *p* = 0.09) and those who were less Open-to-Experience (β = −0.16, *t* = 1.78, *p* = 0.07) endorsed the idea of commercial conspiracies.

## Discussion

This was very much a pilot study: a first attempt to investigate commercial conspiracy theories. The evidence from other work seems to suggest that people tend to be general, rather than specific, conspiracy theorists in the sense that if they appear to believe in one theory they believe in many (Byford, 2011). Indeed Swami et al. (2011) showed that people who tended to believe in conspiracy theories also endorsed a completely fictitious theory made up for experimental purposes. It is probable that those who endorse commercial conspiracy theories also endorse various other conspiracy theories like scientific, criminal, political, religious, and popular culture conspiracy theories. In this study the alpha for the whole scale was high, indicating that irrespective of the particular type of conspiracy identified, people tended to respond in a similar way. In this sense it may not be that there are unique and distinct findings for studies on commercial conspiracy theories because the same individual difference correlates are implicated in beliefs about all conspiracy theories. However, this thesis merits testing.

There was some support for the hypotheses, though it was not strong or consistent. More left-wing, less religious, poorer and pessimistic people endorsed the frequency of conspiracy theories more frequently. They tended to be more Open-to-Experience as predicted but there were no correlations for Agreeableness. It has been shown that more cynical and marginalised people are more likely to agree with conspiracist ideas. Left wing people are often highly sceptical of business and commercial organisations which may explain that association (Byford, 2011). Similarly it could be that the optimism-pessimism and self-assessed wealth (from rich to poor) scores in this study related to belief in conspiracy because of the established finding that conspiracy theories (of all types) thrive among the alienated, disenchanted and dispossessed (Byford, 2011).

Other similar studies in this area have tended to show that although personality and belief variables have been shown to be significantly related to various specific, mainly event-based, conspiracy theories, the size of the correlations/beta weights tended to be small, suggesting that they account for a relatively small amount of the variance. Thus, Swami et al. (2010) found weak but significant and direct effects of Agreeableness, Political Cynicism and Attitudes to Authority correlates of 9/11 conspiracy theories. The same was true of a study looking at the 7/7 conspiracy theories where correlations between personality and belief variables and conspiracy ideation never exceeded *r* = 0.25 (Swami et al., 2011). A similar set of results was found in a study of conspiracy theories about Amelia Earhart (Swami and Furnham, 2012).

This all begs the question for differential psychologists: what factors, be they ability, preference or motivational factors, actually account for “reasonable” amounts of variance in many conspiracy theories? The psychological studies in this area have not been able to identify any factor or experiences which seem to be able to account for anything over 10% of the common variance. It is possible that a social psychological rather than a differential psychological approach to conspiracy theories is more useful.

This study has a number of self-evident limitations. The sample was neither large nor representative of the population as a whole, so threatening the generalizability of the results. Second, the questionnaire needs both editing and expansion to ensure that a wider range of commercial conspiracy theories is tested. Many of the items were not strictly about conspiracies, such as items 4, 6, 7, and 8, which concern modern day marketing and advertising. It may have been better to address some very specific economic and business conspiracy theories like the idea that food companies are lying about genetically modified crops and planning to try to take control the world's food supply. Considerable work needs to be done to devise a robust and reliable measure of commercial conspiracy theories.

Third, it would have been desirable to obtain more information about the participants as well as their beliefs about a host of issues, particularly their attitudes to other conspiracy theories. Studies in this tradition have not been very successful at identifying factors that account for much (say a quarter or more) of the variance in beliefs in conspiracy stories. It is hoped that this pilot study encourages more empirical research in the area.

### Conflict of interest statement

The author declares that the research was conducted in the absence of any commercial or financial relationships that could be construed as a potential conflict of interest.
